# Preclinical Evaluation of STI-8811, a Novel Antibody–Drug Conjugate Targeting BCMA for the Treatment of Multiple Myeloma

**DOI:** 10.1158/2767-9764.CRC-24-0413

**Published:** 2024-10-11

**Authors:** Aaron D. Springer, Rengang Wang, Jiawei Wang, Qinyi Du, Willie Pi, Austin Q. Nguyen, Xiaoqing Li, Alisher Khasanov, Tong Zhu, Zheng Yan, Yufeng Hong, Heyue Zhou, Yanliang Zhang, Lisa Kerwin, Lingna Li, Henry Ji, Hong Zhang

**Affiliations:** 1 Levena BioPharma, San Diego, California.; 2 Sorrento Therapeutics Inc., San Diego, California.

## Abstract

Treatment for patients with multiple myeloma has experienced rapid development and improvement in recent years; however, patients continue to experience relapse, and multiple myeloma remains largely incurable. B-cell maturation antigen (BCMA) has been widely recognized as a promising target for treatment of multiple myeloma due to its exclusive expression in B-cell linage cells and its critical role in the growth and survival of malignant plasma cells. Here, we introduce STI-8811, a BCMA-targeting antibody–drug conjugate (ADC) linked to an auristatin-derived duostatin payload via an enzymatically cleavable peptide linker, using our proprietary C-lock technology. STI-8811 exhibits target-specific binding activity and rapid internalization, leading to G2/M cell-cycle arrest, caspase 3/7 activation, and apoptosis in BCMA-expressing tumor cells *in vitro*. Soluble BCMA (sBCMA) is shed by multiple myeloma cells into the blood and increases with disease progression, competing for ADC binding and reducing its efficacy. We report enhanced cytotoxic activity in the presence of high levels of sBCMA compared with a belantamab mafodotin biosimilar (J6M0-mcMMAF). STI-8811 demonstrated greater *in vivo* activity than J6M0-mcMMAF in solid and disseminated multiple myeloma models, including tumor models with low BCMA expression and/or in large solid tumors representing soft-tissue plasmacytomas. In cynomolgus monkeys, STI-8811 was well tolerated, with toxicities consistent with other BCMA-targeting ADCs with auristatin payloads in clinical studies. STI-8811 has the potential to outperform current clinical candidates with lower toxicity and higher activity under conditions found in patients with advanced disease.

**Significance:**

STI-8811 is a BCMA-targeting ADC carrying a potent auristatin derivative. We report unique binding properties which maintain potent cytotoxic activity under sBCMA-high conditions that hinder the clinical efficacy of current BCMA-targeting ADC candidates. Beyond disseminated models of multiple myeloma, we observed efficacy in solid tumor models of plasmacytomas with low and heterogenous BCMA expressions at a magnitude and duration of response exceeding that of clinical comparators.

## Introduction

Multiple myeloma is characterized by infiltration and poorly controlled proliferation of terminally differentiated plasma cells (PC; refs. 1–3). Although outcomes for multiple myeloma overall have improved dramatically (4–7), patients with multiple myeloma frequently experience relapses with increasing frequency following each subsequent therapy, and multiple myeloma remains incurable for the majority of patients (8–11).

As multiple myeloma is a malignancy of mature PCs, it is typically accompanied by increasing expression of lineage markers, including B-cell maturation antigen (BCMA). BCMA is expressed exclusively in B-cell lineage cells (12–14), is selectively induced during PC differentiation, and is required for long-term PC survival through engagement with A proliferation–inducing ligand and B cell–activating factor (refs. 12, 15–18). BCMA is absent from naïve and most memory B cells and is not critical for overall B-cell homeostasis (18). In contrast, malignant PCs overexpress BCMA and are dependent on BCMA signaling for growth and survival (12, 17, 19, 20). Membrane BCMA can undergo cleavage by γ-secretase to form a soluble BCMA (sBCMA), reducing BCMA expression on multiple myeloma cells (21). sBCMA levels correlate with disease status, therapeutics response, and overall survival (OS; 14, 22, 23). Moreover, gene and protein expression analysis has identified BCMA as the most selective target for relapsed/refractory multiple myeloma (refs. 17, 24, 25), making it a promising target for immunotherapy.

Several immunotherapeutic approaches have been developed targeting BCMA in multiple myeloma, including mAbs, T-cell bispecific antibodies, chimeric antigen receptor (CAR) T-cell therapies, and antibody–drug conjugates (ADC). Although bispecific antibodies have shown promise (26, 27), they often suffer from short half-lives, can lead to cytokine release syndrome, and their efficacy relies on intact T-cell function, which is often compromised in patients with relapsed/refractory multiple myeloma (27–29). Recently, BCMA bispecific therapies, including teclistamab-cqyv (Tecvayli) and linvoseltamab, are also reported to cause serious toxic issues (2023 American Society of Hematology meeting). CAR T-cell therapies are promising but are frequently associated with cytokine release syndrome and neurotoxicity, and treatment requires costly *ex vivo* procedures for each patient, impeding the wide implementation of the therapy (30). For these reasons, ADCs targeting BCMA represent an attractive alternative immunotherapy for the treatment of multiple myeloma.

Herein, we present STI-8811, a BCMA-targeting ADC carrying a potent auristatin derivative, duostatin (Duo5), linked through an enzymatically cleavable pentaglycine peptide linker. We report unique binding properties of STI-8811, which allows the ADC to maintain potent cytotoxic activity under sBCMA-high conditions representative of patients with advanced disease that have previously hindered clinical efficacy of BCMA-targeting ADCs (31). We observed robust *in vivo* efficacy across multiple myeloma models, with efficacy and duration of response exceeding that of the in-house belantamab mafodotin biosimilar. Additionally, we demonstrated efficacy in heterogenous and low BCMA expression systems as well as large solid tumor models of plasmacytomas. Taken together, these results indicated that STI-8811 is a selective and promising immunotherapeutic drug for the treatment of multiple myeloma.

## Materials and Methods

### Test articles and formulations

The anti-BCMA antibody STI-1260, isotype control respiratory syncytial virus IgG1, and biosimilar antibody of belantamab mafodotin (Blenrep, GlaxoSmithKline), J6M0, were produced by the antibody production group at Sorrento Therapeutics. Syntheses of linker payloads and conjugation of all ADCs were prepared by the chemistry and conjugation groups of Levena Biopharma. The sequence of STI-1260 and the chemical structure and synthesis of the payload linker are described in the patent WO 2022184082. The cysteine reactive payload linker, (Gly_5_)-Duo5, was conjugated to STI-1260, as described below. The sequence of J6M0 was taken from the patent US9273141B2 and conjugated to mc-MMAF, as previously described (24).

### Cell culture

The cell lines used in this study were purchased from the ATCC) or Leibniz Institute DSMZ and were routinely cultured in either RPMI-1640 (Catalog #10-041-CV; Corning) or DMEM/F-12 medium (Catalog #10-090-CV; Corning) supplemented with 10% to 20% FBS (Catalog #MT35011CV; Corning) and maintained at 37°C with 5% CO_2_ in a humidified environment. The purchased cell lines included are NCI-H929 (RRID: CVCL_1600), MM1.R (RRID: CVCL_8794), MM1.S (RRID: CVCL_8792), K-562 (RRID: CVCL_0004), OPM-2 (RRID: CVCL_1625), RPMI-8226 (RRID: CVCL_0014), MDA-MB-468 (RRID: CVCL_0419), U266B1 (RRID: CVCL_0566), MM1.S (RRID: CVCL_8792), Daudi (RRID: CVCL_0008), Ramos (RRID: CVCL_0597), ARH-77 (RRID: CVCL_1072), SK-BR-3 (RRID: CVCL_0033), SU-DHL-1 (RRID: CVCL_0538), HDLM-2 (RRID: CVCL_0009), and L-540 (RRID: CVCL_1362). RPMI8226-GFP-Fluc and K562-BCMA were developed internally by stable transfection of GFP-Fluc or BCMA. All cell lines were confirmed *Mycoplasma*-free prior to storage in liquid nitrogen, and experiments were carried out within <15 passages from thawing. Adherent cells (SkBr-3, MDA-MB-468, and SW620) and mixed suspension/slightly adherent cell lines (MM.1R and MM.1S) were harvested by detachment using a cell stripper. Viable cell counts were made by trypan blue exclusion using a Countess or Countess II automated cell counter.

### Conjugation method

STI-1260 antibody was prepared in C-lock buffer [50 mmol/L sodium phosphate and 4 mmol/L ethylenediaminetetraacetic acid (EDTA), pH = 7.2] at 3 to 10 mg/mL. Fresh tris (2-carboxy-ethyl)-phosphin-HC solution was added to give 20× molar excess relative to the antibody and incubated at 37°C for 2 hours or 4°C overnight. Tris (2-carboxy-ethyl)-phosphin-HC was removed using Amicon spin columns, and the reduced antibody was reacted with C-Lock-Gly5-Duo5 at 4 to 6× molar excess at room temperature for >1.5 hours. The final product was purified by preparative size-exclusion chromatography.

### Animals

Female SCID beige mice, 6 weeks of age, were purchased from Charles River Laboratories. Upon receiving, groups of five mice were housed per cage in a controlled vivarium environment and allowed to acclimate for 72 hours prior to experimentation. Rodent chow and water were provided *ad libitum*. The study was conducted following Institutional Animal Care and Use Committee–approved protocols and were performed in the vivarium at Sorrento Therapeutics Inc., which was managed by Explora BioLabs.

### Surface plasmon resonance binding

Kinetic interactions between the antibodies and his-tagged antigen proteins were measured at 25°C using the Biacore T200 surface plasmon resonance system (GE Healthcare). Anti-human fragment crystallizable region antibody was immobilized on a CM5 sensor chip to approximately 8,000 resonance units using the standard N-hydroxysuccinimide/N-Ethyl-N′-(3-dimethylaminopropyl) carbodiimide hydrochloride coupling methodology. The STI-1260 antibody (3 μg/mL) was captured for 60 seconds at a flow rate of 10 μL/minutes. The his-tagged BCMA protein (Catalog #BCA-H522y, ACROBiosystems) was run at seven different dilutions in a running buffer of 0.01 mol/L HEPES, pH 7.4, 0.15 mol/L NaCl, 3 mmol/L EDTA, and 0.05% v/v surfactant P20 (HBS-EP^+^). The 25 nmol/L BCMA-his run was measured two times. All measurements were conducted in HBS-EP^+^ buffer with a flow rate of 30 μL/minutes. A 1:1 (Langmuir) binding model was used to fit the data. Capture time: 60 seconds at a flow rate of 10 μL/minutes. Association time: 120 seconds at a flow rate of 30 μL/minutes. Dissociation time: 300 seconds at a flow rate of 30 μL/minutes.

### Cell-based binding assay

Cells were plated in 96-well round bottom plates (Catalog #3595, Corning) at a density of 1e+5 cells/well in 50 μL FACS buffer (PBS, 2% FBS, and 5 mmol/L EDTA). Each well was treated accordingly with 50 μL of 2× working solutions of test articles prepared in FACS buffer and incubated on ice for 1 hour. Following treatment, the cells were washed and labelled with R-PE–conjugated goat anti-human antibody (Catalog #109-115-098, Lot #141758, Jackson ImmunoResearch, RRID: AB_2337675) diluted 1:250 in FACS buffer for 1 hour on ice, protected from light. The cells were then washed and immediately analyzed using an iQue Intellicyt flow cytometer.

### Internalization assay by fluorescence live-cell imaging and flow cytometry

NCI-H929 and HDLM-2 cells were seeded at a density of 1e+4 cells/well in growth media (50 μL) into 96-well white wall clear bottom plates (Catalog #3603; Corning) coated with poly-L-ornithine solution (Catalog #P4957-50ML, Sigma-Aldrich) and maintained at 37°C for 2 to 4 hours to allow for cell adhesion. Test articles were prepared at 2× final concentration (20 μg/mL) and labelled with IncuCyte FabFluor-pH Red Antibody Labeling Reagent (Catalog #4722, IncuCyte) at a 3:1 molar ratio for 15 minutes protected from light. FabFluor-labelled test articles (50 μL/well) were then added to the cells, and the plate was immediately transferred into Sartorius IncuCyte S3 Live Cell Analysis System (RRID: SCR_023147). Phase-contrast and red fluorescence images were obtained in duplicates or triplicates per well every 30 minutes for 48 hours. Images captured using the IncuCyte Zoom system were analyzed using IncuCyte software (version 2016B).

For flow cytometry measurement, NCI-H929 cells were seeded at a density of 1e+5 cells/well in 50 μL growth media, treated with test articles labelled with Alexa Fluor 647 (AF647), and incubated on ice for 1 hour to allow binding saturation before incubation at 37°C. At each timepoint, cells were pelleted, washed, and incubated with proteinase K (250 μg/mL in PBS; Catalog #AM2546; Thermo Fisher Scientific) at 37°C for 10 minutes to remove surface-bound protein. After wash and resuspension with FACS buffer (150 μL), the cells were immediately analyzed using an Attune NxT flow cytometer (Thermo Fisher Scientific, RRID: SCR_019590). Quantum AF647 molecules of equivalent soluble fluorophore beads (Catalog #647; Bang Laboratories) were used for fluorescence quantitation. Molecules of equivalent soluble fluorophore values were converted to antibodies bound per cell by normalization to the known fluorophore:mAb ratio.

### Cell cytotoxicity assay

All cells were seeded into 384-well white wall clear bottom plates (Catalog #3765; Corning) at a density of 2,500 cells/well in growth media. Cell treatment was performed in either technical triplicates or duplicates and maintained at 37°C for a 120-hour assay. For sBCMA spike-in experiments, NCI-H929 and OPM2 cells were treated with listed treatment of STI-8811 in the presence of a 2-fold dilution curve of sBCMA (Catalog #BCA-H522y, ACROBiosystems) and otherwise treated as listed above. After treatment, cell viability was determined by CellTiter-Glo 2.0 assay (Catalog #G9243; Promega) based on the manufacturer’s instruction. Luminescence was measured using a Tecan SPARK multimode microplate reader (Tecan, RRID: SCR_021897).

### Receptor quantification measurement

Cells were plated at a density of 1e+5 cells/well in 50 μL of FACS buffer into a 96-well U-bottom plate. Fluorescently labelled STI-1260-AF647 was prepared, and the cells were treated with 50 μL of working solution of mAb at 4°C for 1 hour at a final concentration of 10 μg/mL. The cells were then washed and analyzed by flow cytometry. Alexa Fluor 647 quantification beads (Catalog #647, Bangs Laboratories, Inc.) were run in parallel to allow for receptor quantification, as described above.

### Cell-cycle analysis

NCI-H929 and K562 cells were plated at a density of 3e^+5^ cells/well in 3 mL complete media into a 6-well plate. Each well was treated accordingly with 10× working solutions of test reagents prepared in culture medium to a final concentration of 1 nmol/L per sample. After 72 hours of treatment, the cells were harvested and fixed with ice-cold 70% (v/v) ethanol/dH_2_O on ice for at least 15 minutes prior to storage at −20°C. For sample staining and analysis, plates were removed from −20°C and washed and resuspended in FxCycle Propidium Iodide (PI)/RNAse Staining Solution (Catalog #F10797; Thermo Fisher Scientific) for 20 minutes at room temperature, protected from light. PI incorporation was measured using an Attune NxT flow cytometer (Thermo Fisher Scientific).

### Apoptosis assay

NCI-H929 and K562 cells were plated into a 384-well white flat bottom plate (Catalog #3765, Corning) at a density of 5,000 cells/well in 12.5 μL. The cells were treated by adding 12.5 μL of 2× working solution and incubated for 48 hours at 37°C with 5% CO_2_. Docetaxel (10 nmol/L ≈ 8 μg/mL) was used as a positive control for robust caspase-3 and -7–dependent activation of apoptosis. Caspase activity for apoptosis detection was determined using the Caspase-Glo 3/7 assay (Catalog #G8091; Promega) based on the manufacturer’s instruction. Luminescence was measured using a TECAN Spark plate reader (Tecan).

### Bystander assay

K562 cells were labelled with carboxyfluorescein diacetate succinimidyl ester (cFSE; Catalog #C34554, Invitrogen) following the manufacturer’s instruction. cFSE-labelled K562 cells were pelleted and resuspended in growth media prior to use in bystander assay. NCI-H929 cells and cFSE-labelled K562 cells were plated in a 96-well U-bottom plate (Catalog #3879; Corning) at a density of 5e+4 total cells/well (4e+4 NCI-H929 cells/well, 1e+4 cFSE-K562 cells/well) in growth media. Cell treatments were performed in technical quadruplicates over a 72-hour assay and maintained at 37°C. After 72 hours of treatment, the cells were washed with staining buffer (10 mmol/L HEPES pH 7.4, 140 mmol/L NaCl, 2.5 mmol/L CaCl_2_) and stained with Brilliant Violet 421 Annexin V (Catalog #640924, BioLegend) and PI (Catalog #P4864, Sigma-Aldrich) for 15 minutes at room temperature, protected from light. The cells were analyzed immediately by flow cytometry using an Attune NxT instrument.

### Serum stability of STI-8811

Plasma was thawed and centrifuged at 3,000 *g* for 5 minutes to remove any precipitate. STI-8811 was diluted to 0.1 mg/mL in human, monkey, or rat plasma or 5% BSA in PBS, followed by sterile filtration (Millex-GP, Millipore Catalog No. SLGP033RB). Triplicate timepoint aliquots were taken at 0, 1, 2, 4, 7, 14, and 21 days of incubation and stored at −80°C until Duo5 concentration analysis by LC-MS/MS.

### Anticancer efficacy evaluated in animal models

Cell lines were cultured as previously described for a period of 2 to 3 weeks before harvesting for implantation. A measure of 2 to 5 million cells/100 μL of PBS (Catalog #21-040-CV, Corning) and Matrigel (Catalog #354234, Corning) 1:1 (v/v) mixture were implanted to the right upper flank of each mouse by s.c. injection.

Tumor volume (*TV*) measurement was started at day 14 after tumor cell inoculation and was performed twice weekly using a digital caliper (Catalog #62379-531, VWR).

*TV* was calculated as follows:

$$TV\;=\;\left[\mathrm{length}\;\times \;{\left(\mathrm{width}\right)}^{2}\right]/2$$

where length is the longest longitudinal diameter and width is the widest transverse diameter.

Mice were randomly assigned to each treatment group and were included based on the presence of a palpable tumor at the time of treatment.

In the RPMI-8226-Fluc mouse model, tumor burden was measured by bioluminescence intensity determined by the PerkinElmer IVIS Spectrum *In Vivo* Imaging System (RRID: SCR_018621; ref. 32).

All compounds were diluted in PBS to working concentrations which were calculated according to treatment regimens, and a volume of 0.2 mL was injected per mouse.

### Preclinical pharmacokinetic studies in mice and monkeys

Pharmacokinetics (PK) of STI-8811 and STI-1260 were investigated via a single i.v. administration at 8 mg/kg in SCID beige mice. In cynomolgus monkeys, STI-8811 was given via i.v. injection at 0.25, 0.75, and 2.25 mg/kg. The concentrations of ADC and total antibody (TAb) of STI-8811 and STI-1260 antibodies in mouse and cynomolgus monkey serum/plasma samples were measured by validated ELISA methods. The concentration of Duo5 was measured using a LC-MS/MS assay. Immunogenicity (anti-drug antibody) was detected based on a bridging ELISA assay with Molecular Devices/Spectra Max plus 384. All data were acquired and analyzed by SoftMax Pro 7.0 or Phoenix WinNonlin v8.1 software.

### Preclinical safety and toxicokinetic studies

The pivotal toxicity study in nonhuman primates were performed by InnoStar Bio-tech Nantong Co., Ltd. in compliance with the animal welfare policies and guidelines approved by the National Medical Products Administration of China for toxicity and PK studies.

### Statistical analysis

All experiments were conducted in duplicates or triplicates. Data are presented as the mean ± SD. Statistical analysis was performed by the Student *t* test or a one-way/two-way ANOVA with multiple comparisons by GraphPad Prism (RRID: SCR_002798). *P* < 0.05 was considered statistically significant.

### Data availability

The data generated in this study are available upon request from the corresponding author.

## Results

### STI-8811 binds specifically to BCMA and shows target-dependent internalization in multiple myeloma cell lines

**Figure 1:**
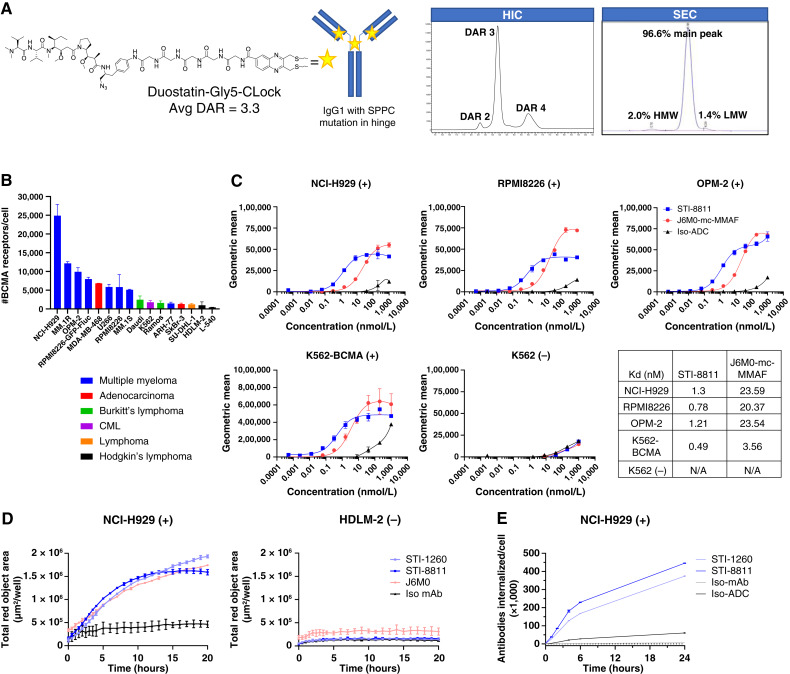
STI-8811 structure, binding, and internalization capacity in BCMA-expressing tumor cell lines. **A,** Structure and analytical data of STI-8811 showing cysteine rebridging conjugation, cleavable Gly5 linker, and Duo5 payload. **B,** BCMA surface expression in 16 tumor cell lines (*n* = 2–4) determined by quantitative flow cytometry using STI-1260 labelled with Alexa Fluor 647. Receptor binding signal was normalized to the flurophore/mAb ratio, and quantification was performed by reference to a standard bead curve. **C,** Binding of STI-8811, J6M0-mc-MMAF, and their corresponding mAbs (*n* = 3) to BCMA-positive NCI-H929, RPMI8226, and OPM-2; BCMA-negative K562; and BCMA-overexpressing K562-BCMA cell lines. **D,** Live-cell imaging internalization assay in BCMA-positive NCI-H929 (left) and BCMA-negative HDLM-2 (right) cell lines (*n* = 3). **E,** Quantitative internalization of STI-1260 (light blue) and STI-8811 (dark blue) measured under saturating antibody conditions (67 nmol/L) by flow cytometry in the NCI-H929 cell line. All data are represented as the mean ± SD. Avg DAR, average drug to antibody ratio; CML, chronic myeloid leukemia; HIC, hydrophobic interaction chromatography; HMW, high molecular weight; LMW, low molecular weight; SEC, size exclusion chromatography; SPPC, hinge mutation from wild type CPPC amino acid hinge sequence.

The structure and analytical data of STI-8811 are shown in Fig. 1A. Target-dependent selective binding is vital for ADC therapeutic efficacy and safety. We first analyzed the cell-binding specificity of an unconjugated anti-BCMA antibody, STI-1260, to multiple myeloma cell lines with varying BCMA expressions (Fig. 1B). Binding of STI-1260 required BCMA expression and was target-specific and dose-dependent (*K*_*d*_, 1.16–1.53 nmol/L; Fig. 1C). Binding was not observed in the BCMA-negative cell line K562, although binding was restored following exogenous BCMA overexpression (K562-BCMA).

To benchmark STI-1260, we produced a biosimilar of the antibody used in belantamab mafodotin. The amino acid sequence of this antibody (J6M0) was taken from patent literature and produced in a Chinese hamster ovary expression system, providing a fully glycosylated J6M0 biosimilar. Notably, clinical belantamab mafodotin is afucosylated in order to enhance antibody-dependent cellular cytotoxicity. However, this afucosyl modification does not alter the binding nor the cytotoxic potency of the J6M0 antibody (24). Binding of our J6M0 biosimilar to multiple myeloma cell lines revealed a relatively higher *K*_*d*_ (15.3–33.2 nmol/L) compared with our STI-1260 antibody.

Conjugation of payload and linker to each antibody to produce STI-8811 and J6M0-mcMMAF resulted in similar *K*_*d*_ of 0.78 to 1.30 nmol/L and 20.4 to 23.6 nmol/L, respectively. No significant difference was observed between the antibodies and their corresponding ADCs, confirming that conjugation of the payload and linker had minimal impact on the binding characteristics of STI-1260.

Internalization is critical for efficient linker cleavage and payload release. Analysis of antibody internalization by real-time fluorescent microscopy revealed that both STI-8811 and the unconjugated STI-1260 antibody were efficiently internalized into BCMA-positive NCI-H929 cells at a comparable rate and magnitude to the benchmark antibody J6M0 (Fig. 1D). Quantitative analysis of internalization into NCI-H929 cells by flow cytometry revealed a rapid initial (0–4 hours) internalization of 3.2e+4 antibodies/hour and 6.0e+4 ADC/hour followed by a slower, steady-state internalization rate of 1.2e+4 antibodies/hour and 1.3e+4 ADC/hour for STI-1260 and STI-8811, respectively (Fig. 1E). Internalization into BCMA-medium cell line RPMI8226 revealed a similar pattern but at reduced magnitude, concomitant with the lower overall BCMA expression (Supplementary Fig. S1). Taken together, STI-8811 bound efficiently to BCMA and was internalized by multiple myeloma cell lines.

### STI-8811 kills multiple myeloma cell lines in a BCMA-dependent manner

**Figure 2:**
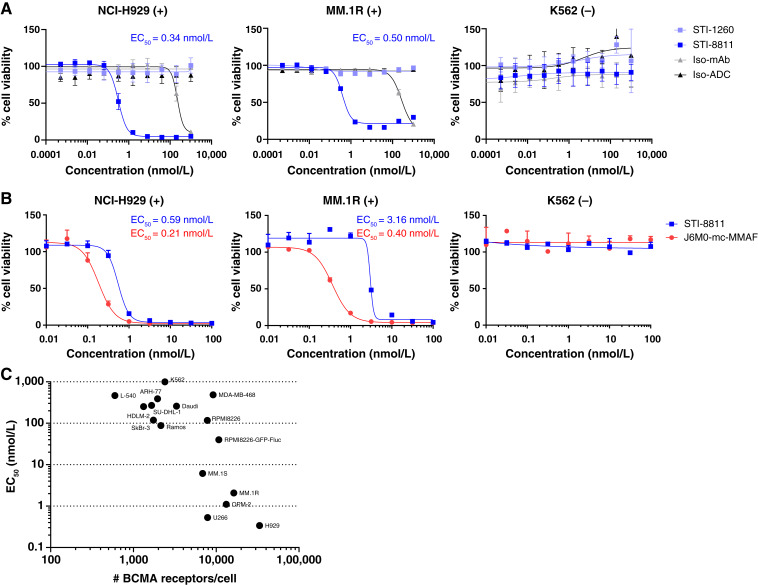
Cytotoxic activity of STI-8811 on multiple myeloma cell lines and correlation with BCMA surface expression. **A,** Cytotoxic activity of STI-8811 in indicated multiple myeloma cell lines after 120 hours of incubation (*n* = 3–9). Data are represented as the mean ± SD. **B,** Comparison of cytotoxic activity between STI-8811 and the belantamab mafodotin biosimilar (J6M0-mc-MMAF) after 120 hours of incubation. **C,** Correlation between the EC_50_ value and BCMA surface expression on 16 tumor cell lines. EC_50_, treatment concentration at 50% signal.

We examined the cytotoxic activity of STI-8811 following 120 hours treatment in BCMA-positive multiple myeloma cell lines NCI-H929 and MM.1R as well as BCMA-negative cell line K562 (Fig. 2A). Treatment with STI-8811, but not the control groups (STI-1260, isotype mAb, or isotype ADC), induced cytotoxicity in NCI-H929 and MM.1R in a dose-dependent manner with subnanomolar EC_50_. Comparison to J6M0-mcMMAF revealed a 2- to 8-fold lower potency for STI-8811 in the positive cell lines, with greater difference in mid-low–level BCMA cell lines (Fig. 2B). Potency of our J6M0-mcMMAF biosimilar was consistent with published cytotoxicity in these same cell lines, validating our biosimilar benchmark compound (24).

We further examined a panel of 16 cancer cell lines covering a wide range of tumor origin and BCMA expressions (Supplementary Table S1). A comparison of BCMA expression and sensitivity to STI-8811 treatment revealed a threshold of BCMA expression required for activity (∼2,000 receptors/cell), above which we observed an inverse correlation between receptor expression and EC_50_, consistent with BCMA-mediated ADC cytotoxicity (Fig. 2C).

### STI-8811 induces apoptosis through G2/M arrest and caspase 3/7 activation

**Figure 3:**
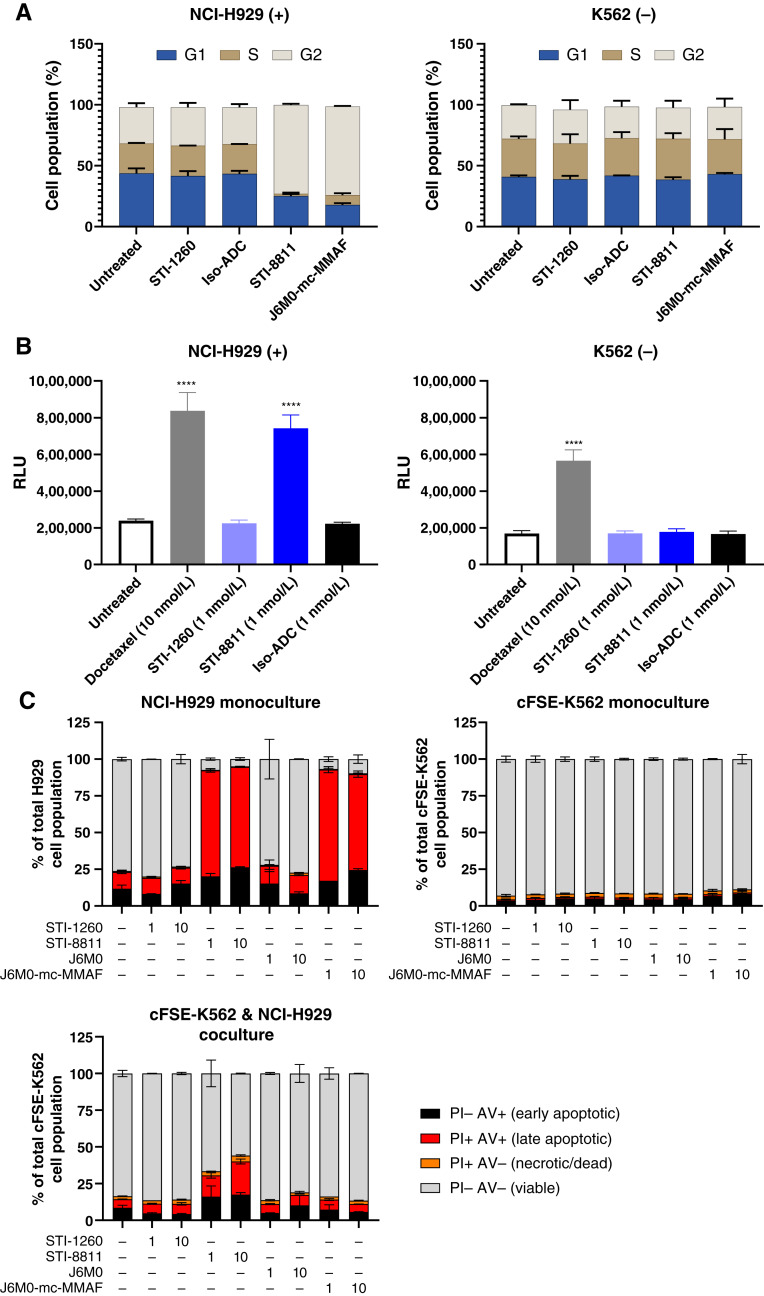
Mechanism-of-action of STI-8811. **A,** Cell-cycle assay in NCI-H929 (left) and K562 (right) cell lines following 72 hours of treatment at 1 nmol/L. Bar height showing the percentage of cell population in each cell-cycle phase (*n* = 2). **B,** Caspase 3/7 apoptosis reporter assay on NCI-H929 (left) and K562 (right) cell lines after 48 hours of treatment. Bar height indicating the level of caspase 3/7 activation measured by relative light units (*n* = 4), ****, *P* < 0.0001. Data were analyzed using one-way ANOVA. **C,** Bystander assay showing the percentage cell population in each stage of apoptosis as measured by annexin V binding and PI staining after 72 hours of incubation. NCI-H929 monoculture (left), cFSE-labelled K562 (cFSE-K562) monoculture (middle), and cFSE-K562 viability in coculture with NCI-H929 (left; *n* = 2). Av, annexin V; RLU, relative light unit.

Microtubule inhibitors, including auristatins, induce cell-cycle arrest at G2/M. To confirm the mechanism of action of our auristatin-derived Duo5 payload, we analyzed cell-cycle changes following STI-8811 treatment. Cell-cycle arrest at G2/M was observed for STI-8811 in BCMA-positive multiple myeloma cell line NCI-H929 but not in BCMA-negative K562 (Fig. 3A). Furthermore, we confirmed that BCMA-positive cell lines exhibited caspase 3/7–dependent apoptosis upon treatment with STI-8811 (Fig. 3B). No cell-cycle arrest or caspase 3/7 activity was seen following treatment with either isotype ADC or parental STI-1260 antibody. Taken together, the mechanism of action for STI-8811 is consistent with auristatin payloads and proceeds via Duo5-dependent G2/M arrest followed by caspase 3/7 activation and apoptosis.

### STI-8811 induces a potent bystander effect *in vitro* in heterogenous BCMA expression models

A subset of patients receiving BCMA-targeted immunotherapy develop multiple myeloma relapse with low or absent BCMA expression, suggesting immunoediting after initial treatment (30). Additionally, as much as 34% of patients with multiple myeloma present with solid tumor plasmacytomas (33–35). Under these solid or heterogenous tumor conditions, ADCs with membrane-permeable payloads have advantages in gaining access to adjacent negative tumor cells via the “bystander effect.” To demonstrate the bystander activity of STI-8811, we cocultured BCMA-positive NCI-H929 and BCMA-negative K562 cell lines, treated with STI-8811 for 72 hours, and measured cytotoxicity in each cell line by flow cytometry. As expected, treatment with STI-8811 induced cytotoxicity in NCI-H929 but not K562 monoculture (Fig. 3C). In contrast, treatment of NCI-H929 and K562 coculture resulted in cytotoxicity in the NCI-H929 cell line and bystander cytotoxicity in the K562 cell line. We also tested J6M0-mcMMAF and found minimal cytotoxicity in K562 cells under coculture conditions, consistent with the lack of bystander activity of MMAF (24). These results demonstrated that STI-8811 elicits a bystander effect and could potentially provide improved treatment outcomes for patients with multiple myeloma harboring heterogeneous BCMA expressions or solid plasmacytomas.

### STI-8811 maintains *in vitro* activity in the presence of sBCMA

**Figure 4:**
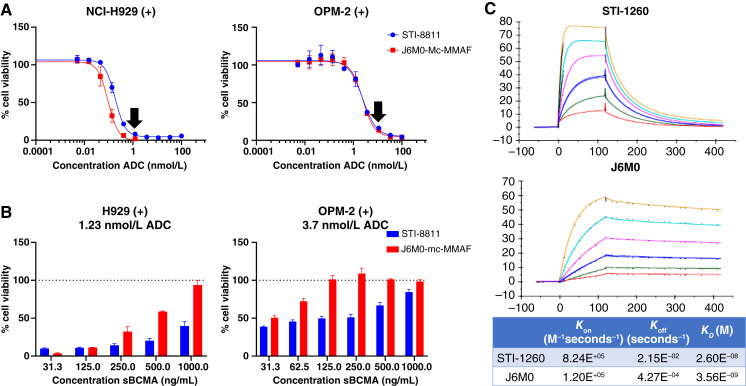
Effect of sBCMA on the cytotoxic activity of STI-8811 and J6M0-mc-MMAF. **A,** Cell viability of NCI-H929 (left) and OPM-2 (right) after treatment with serial dilutions of STI-8811 and J6M0-mc-MMAF in the absence of sBCMA (*n* = 3). Arrows represent the ADC dose that achieves 80%–90% cell death. Data are represented as the mean ± SD. **B,** Cell viability of NCI-H929 (left) and OPM-2 (right) after treatment with STI-8811 and J6M0-mc-MMAF at 1.23 nmol/L (left) or 3.7 nmol/L (right) in the presence of a sBCMA dilution curve (*n* = 3). Data are represented as the mean ± SD. **C,** Surface plasmon resonance assay showing the binding kinetics of STI-1260 and J6M0 mAb.

In patients with multiple myeloma, plasma sBCMA levels correlate with disease progression, treatment response, and OS (22, 23). In healthy individuals, sBCMA is typically <83 ng/mL with a median of 36.8 ng/mL, whereas in patients with multiple myeloma, plasma sBCMA is elevated to 100 to 1,000 ng/mL (23, 36–38). To evaluate the potential impact of sBCMA on STI-8811 activity, we monitored the cytotoxicity of STI-8811 under conditions correlated with sBCMA levels in patients with advanced relapsed/refractory multiple myeloma. We first determined the dosage required to achieve 80% to 90% cytotoxicity for both STI-8811 and J6M0-mcMMAF in NCI-H929 and OPM-2 cell lines (Fig. 4A). The rationale behind using this fixed dose is that it provides a robust cytotoxicity starting point that is similar in magnitude between both test articles while remaining close to the steepest part of the dose curve, providing sensitivity to small perturbations in cytotoxicity as a result of sBCMA addition. Then we subsequently treated these cell lines with the predetermined fixed concentration of STI-8811 or J6M0-mcMMAF under increasing sBCMA concentrations (Fig. 4B). We observed that STI-8811 activity in NCI-H929 cells decreased only slightly as sBCMA concentration increased, with >60% of activity remaining as sBCMA concentration reached 1,000 ng/mL; in contrast, J6M0-mc-MMAF cytotoxic activity was reduced to less than 10% of the maximum response under the same condition. Similar results were observed in the BCMA-mid OPM-2 cell line, in which J6M0-mcMMAF lost all activity at 125 ng/mL sBCMA, whereas STI-8811 retained activity at 1,000 ng/mL sBCMA. These results indicated that, unlike J6M0-mcMMAF, the cytotoxic activity of STI-8811 was largely retained at sBCMA concentrations seen in patients with advanced relapsed/refractory multiple myeloma and thus may be less affected by BCMA shedding in the clinical settings, allowing for reduced dosing.

To address the mechanism behind the differential impact of sBCMA on the cytotoxicity of STI-8811 and J6M0-mcMMAF, we analyzed the binding kinetics of both antibodies by surface plasmon resonance. The association rate of STI-1260 was 8.24e+5 1/millisecond, the dissociation rate was 2.15e−2 1/second, and the *K*_*d*_ was 26 nmol/L (Fig. 4C); the association rate of J6M0 was 1.20e+5 1/millisecond, the dissociation rate was 4.27e−4 1/second, and the *K*_*d*_ was 3.56 nmol/L, consistent with the reported values in the literature (24). These results demonstrated that STI-1260 has a distinct binding profile to BCMA with a unique fast-on/fast-off rate that may resist sequestration by sBCMA present in the blood and differentiates itself from current clinical ADCs.

### STI-8811 eradicates tumors in xenograft models

**Figure 5:**
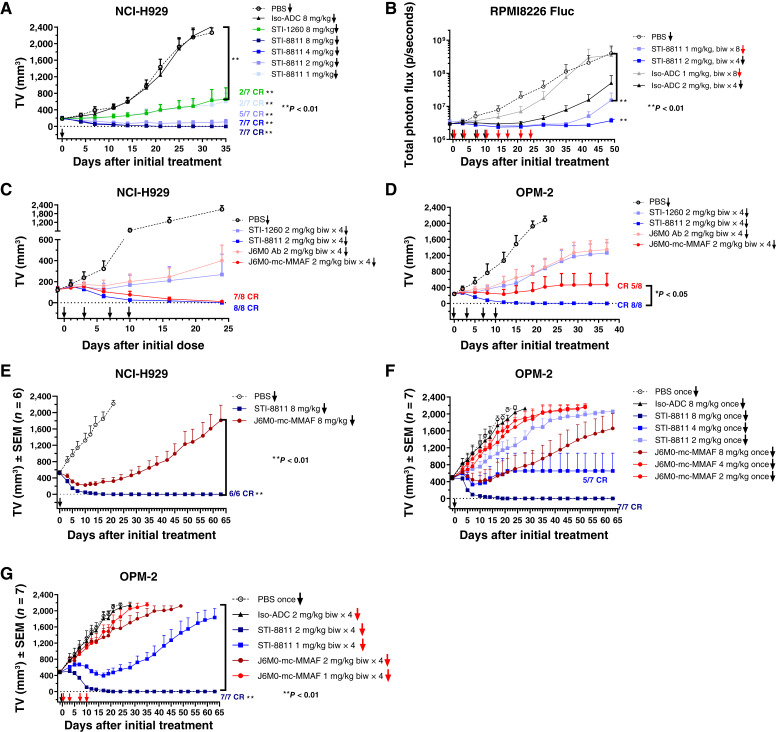
Tumor suppression study of STI-8811 in xenograft mouse models. **A,** Dose escalation study of STI-8811 in NCI-H929 s.c. xenograft tumor models. Mice (*n* = 7) were treated i.v. with single dose of STI-8811 or PBS as a control. All data are represented as the mean ± SD. **B,** Study of STI-8811 in a disseminated RPMI8226 tumor model expressing firefly luciferase (RPMI8226-GFP-Fluc). Mice (*n* = 7–8) were treated i.v. biweekly with four or eight doses. **C,** Comparison of tumor suppression resulting from repeated treatments of STI-8811 and J6M0-mc-MMAF in NCI-H929 and (**D**) OPM-2 tumor models. Mice (*n* = 8) were treated i.v. with four doses of indicated molecules at a biweekly regimen. **E,** Comparison of single-dose treatment of STI-8811 and J6M0-mcMMAF in large (∼500 mm^3^) NCI-H929 and (**F**) large OPM-2 tumor models. Mice (*n* = 6) were treated i.v. with a single dose of ADCs or the PBS control. **G,** Comparison of repeated-dose treatment of STI-8811 and J6M0-mcMMAF in a large OPM-2 tumor model. Mice (*n* = 7) were treated i.v. biweekly with four doses. biw, biweekly, CR, complete response.

The antitumor activity of STI-8811 was determined *in vivo* using NCI-H929 and OPM-2 s.c. xenograft models in SCID beige mice and RPMI8226-GFP-Fluc disseminated xenograft model in NOD/SCID gamma mice. Figure 5A shows the inhibition of tumor growth following a single dose of STI-8811 at different dose levels in the NCI-H929 xenograft model. Over the course of the 35-day study, a single dose of 1 mg/kg STI-8811 inhibited *TV* by 80%, with two tumors being completely eradicated (*P* < 0.01, vs. PBS control). This effect was also observed with 8 mg/kg of the parental antibody, STI-1260, but not with the isotype control ADC. A dose of 2 mg/kg STI-8811 eradicated five of seven tumors and reduced the size of the remaining two tumors (*P* < 0.01, vs PBS control), whereas a single injection of STI-8811 at 4 or 8 mg/kg eliminated all tumors by day 21, and no recurrence was noted during the entire 35-day study period. No body weight loss was observed in any of the STI-8811–treated mice at doses up to 8 mg/kg. In a separate study, repeated administration of STI-8811 at 2 mg/kg twice weekly for 2 weeks also completely eradicated all NCI-H929 tumors in mice for more than 60 days (Supplementary Fig. S2).

RPMI8226 is a multiple myeloma tumor cell line that expresses a moderate level of BCMA compared with NCI-H929. In the disseminated RPMI8226-GFP-Fluc xenograft model, tumor cells were intravenously implanted into NOD/SCID gamma mice, allowing the tumor burden to be distributed throughout the body, mimicking the diffuse nature of multiple myeloma in patients. STI-8811 was administered 20 days after RPMI8226-GFP-Fluc implantation when the tumor burden signal reached 3 × 10^6^ p/seconds. Tumor burden in STI-8811–treated mice decreased below baseline levels for up to 35 days after eight repeated doses at 1 mg/kg or up to 42 days after four repeated doses of 2 mg/kg (*P* < 0.01, vs. PBS control; Fig. 5B). No body weight loss was observed.

The ability of STI-8811 to eradicate BCMA-expressing tumors was further investigated and benchmarked against J6M0-mcMMAF in both NCI-H929 and OPM-2 xenograft models under repeat dosing regimens. We showed here that four repeated doses of 2 mg/kg STI-8811 eliminated all NCI-H929 tumors, whereas J6M0-mcMMAF eradicated seven of eight tumors under the same regimen (Fig. 5C). The enhanced efficacy of STI-8811 over J6M0-mcMMAF was further demonstrated in OPM-2 xenografts expressing BCMA at a moderate level. STI-8811 completely eliminated tumors after repeated doses of 2 mg/kg (*P* < 0.05, vs. J6M0-mc-MMAF), whereas J6M0-mcMMAF showed complete eradication in five of eight tumors (Fig. 5D).

Although multiple myeloma is often described as a “liquid” tumor, a subset of patients develops solid soft-tissue plasmacytomas, measuring 1 to 7.5 cm, originating from adjacent bone lesions or in extramedullary soft tissues and organs (33–35). To evaluate the therapeutic activity of STI-8811 against large solid multiple myeloma tumors, NCI-H929 and OPM-2 tumors were grown subcutaneously in SCID beige mice to an average size of ∼500 mm^3^ prior to treatment. A single dose of 8 mg/kg STI-8811 resulted in complete tumor eradication by 21 days in all mice harboring large NCI-H929 tumors with no regrowth during the entire 63-day study period (*P* < 0.01, vs. J6M0-mc-MMAF), whereas the same dose of J6M0-mcMMAF resulted in only modest tumor regression, followed by rapid tumor regrowth (Fig. 5E). In BCMA-mid OPM-2 tumors, the same single 8 mg/kg dose of STI-8811 also eliminated all large tumors without regrowth, whereas no tumor eradication was observed in any of the J6M0-mcMMAF–treated mice (Fig. 5F). In a repeated dose study in the OPM-2 model, four repeated doses of STI-8811 at 2 mg/kg were sufficient to completely eradicate all large tumors within 3 weeks without rebound (*P* < 0.01, vs J6M0-mc-MMAF), whereas J6M0-mcMMAF showed almost no tumor growth inhibition (Fig. 5G). Overall, STI-8811 maintained greater tumor regression/eradication and duration of response compared with J6M0-mcMMAF regardless of tumor size and BCMA expression.

### Plasma stability of STI-8811

We assessed the plasma stability of STI-8811 by incubating the ADC with human, rat, and cynomolgus monkey plasma for 21 days at 37°C (*n* = 3). After each incubation time point, free payload was measured by LC-MS/MS. After 21 days of incubation, free payload increased to a maximum of 0.34% in human plasma, 0.22% in monkey plasma, and 0.83% in rat plasma (Table 1). We concluded that STI-8811 is stable in plasma with minimal payload release.

**Table 1:** Serum stability of STI-8811 in rat, cynomolgus monkey, and human serum determined by the percentage of payload release over 21 days (*n* = 3)

Species	Concentration tested	% payload released
Rat	100 μg/mL	0.83%
Cynomolgus monkey		0.22%
Human		0.54%
0.5% BSA PBS		0.13%

Payload concentration was measured by LC-MS/MS and converted to the percentage of the total ADC payload.

### PK study in SCID beige mice and cynomolgus monkeys

**Figure 6:**
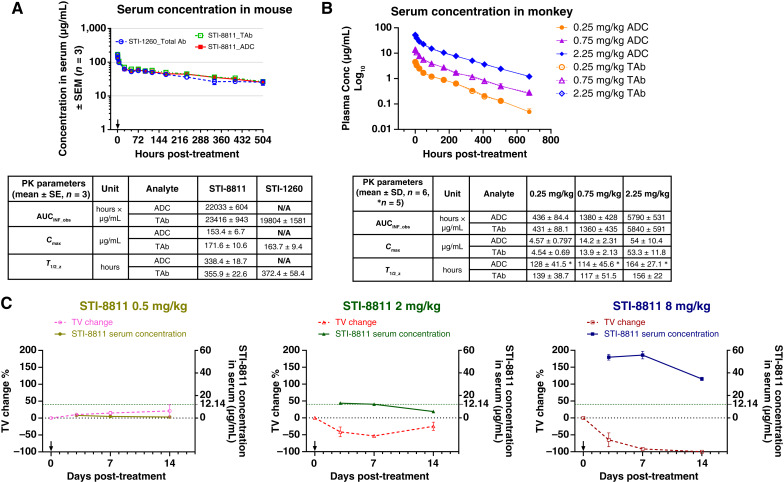
Serum stability and PK studies. **A,** PK curves of STI-8811 and STI-1260 in SCID beige mice following single-dose 8 mg/kg i.v. administration. **B,** PK curves of STI-8811 and STI-1260 in cynomolgus monkeys with single-dose i.v. administration. **C,** Serum concentration vs. *TV* change of STI-8811 in a NCI-H929 xenograft model in SCID beige mice over 14 days (*n* = 3). Data are represented as the mean ± SD.

The PK of STI-8811 and STI-1260 were investigated in SCID beige mice after i.v. administration at 8 mg/kg (Fig. 6A). Serum concentrations of TAb and ADC were measured by ELISA. TAb and ADC concentrations were quantifiable up to 504 hours post-dose. Peak serum concentrations (*C*_max_) were observed between 0 and 0.25 hours post-dose. The *C*_max_ (mg/mL) of TAb and ADC were 171.6 ± 10.6 and 153.4 ± 6.7 and their AUC_inf_obs_ (hours × µg/mL) were 23,416 ± 943 and 22,033 ± 604, respectively. The PK profiles for intact ADC and TAb were similar to each other and comparable with those of the unconjugated antibody STI-1260, indicating that minimum payload was released from the antibody in circulation and that conjugation of the Duo5 payload did not alter the PK properties of STI-1260 in mice.

In the PK study in cynomolgus monkeys, following a single i.v. injection of STI-8811 at doses of 0.25, 0.75, or 2.25 mg/kg, the exposure of STI-8811 (*C*_max_ and AUC) was approximately dose proportional (Fig. 6B). At all dose levels, peak plasma concentrations of ADC and TAb were observed between 0 and 0.25 hours post-dose. The mean plasma half-life (*T*_1/2_) was 114 to 164 hours for ADC and 117 to 156 hours for TAb. No gender difference was observed. The major PK parameters of STI-8811 TAb and ADC in cynomolgus monkey plasma are shown in the table of Fig. 6C. Because the concentration of Duo5 in all samples was below the lower limit of quantification (0.200 ng/mL), no PK parameters in plasma were estimated. The PK profiles of the intact ADC and TAb for STI-8811 were similar to each other and comparable with those of the unconjugated antibody, STI-1260, confirming that the ADC was stable in microcirculation of a second species and that conjugation of the payload did not alter the PK of the antibody.

To evaluate the relationship between exposure (PK) and efficacy [pharmacodynamics (PD)], NCI-H929 tumor-bearing mice were treated with a single i.v. injection of STI-8811 and monitored over 14 days for both tumor growth as well as serum ADC concentration (Fig. 6C). At a single dose of 0.5 mg/kg, tumor size continued to increase during the study, and the serum ADC concentration remained low. At 2 mg/kg, tumor size reduced on days 3 and 7 but began to grow again on day 14. The corresponding serum ADC concentrations were stable for the first 7 days (13.57–14.13 μg/mL) and declined to 6.40 μg/mL on day 14. In the 8/mg/kg treatment, STI-8811 eliminated all tumors and the ADC serum concentration remained above 34.60 μg/mL throughout the course of study. Taken together, we used the 2 mg/kg dose STI-8811 serum concentration at which the tumors start to decrease (12.14 μg/mL) as a surrogate concentration sufficient for tumor burden reduction in this model. This means that lower serum concentrations than this may result in the regrowth of the tumor.

### Toxicity in cynomolgus monkey

The pivotal toxicity study in cynomolgus monkey resulted in findings consistent with the described mechanism of action for an auristatin-derived Duo5 payload delivery to BCMA-targeted cells. Target organ toxicity was observed in bone marrow (BM) and rapidly dividing cells derived from the hematopoietic system, consistent with those of the auristatin payload MMAE. The target organ toxicity was most prominent in erythroid and myeloid centers of the BM, along with toxicity to the male reproductive organs. Mild single-cell necrosis/mitosis of the corneal epithelium of the eyes was noted in female animals following treatment with STI-8811. No treatment-related effects on cardiovascular, respiratory, and central nervous systems were observed.

## Discussion

Treatment of multiple myeloma has improved dramatically but remains characterized by frequent relapse with increasing clonal heterogeneity and therapeutic resistance. Relapsed/refractory multiple myeloma continues to challenge targeted immunotherapy, and ADCs have emerged as a promising therapy for multiple myeloma and relapsed/refractory multiple myeloma.

BCMA is expressed exclusively in B-cell lineage origins (12, 13), is selectively induced during PC differentiation, and is highly overexpressed in malignant PCs but is not critical for overall B-cell homeostasis (12, 17–20). An anti-BCMA ADC conjugated to MMAF (belantamab mafodotin) was shown to be beneficial in patients with multiple myeloma in clinical studies and became the first BCMA-targeting ADC to receive accelerated approval from the FDA as monotherapy in 2020. However, the approval was withdrawn in 2022 after failing to meet the primary efficacy endpoint of progression-free survival benefit compared with standard chemotherapy in the phase III DREAMM-3 trial (NCT04162210). Here, we outline a novel BCMA-targeting ADC, STI-8811, which binds BCMA with unique fast-on/fast-off binding that maintains specific and efficient internalization into BCMA-expressing multiple myeloma cell lines, resulting in effective tumor eradication both *in vitro* and *in vivo*. These results matched or exceeded the *in vivo* activity of our benchmark ADC, a biosimilar of belantamab mafodotin. Importantly, in all cases in which comparison with this biosimilar were made, the biosimilar displayed comparable activity to published literature values for matched cell lines, mouse models, and experimental parameters (24), providing meaningful preclinical comparison for STI-8811.

Patient sBCMA levels correlate with multiple myeloma disease status, therapeutics response, and OS in multiple myeloma (22, 23). Clinical outcomes of patients treated with an anti-BCMA ADC, AMG-224, correlated with sBCMA levels (31), and ongoing phase III DREAM-5 clinical trials for belantamab mafodotin include cotreatment with a γ-secretase inhibitor to reduce sBCMA levels and increase membrane BCMA expression on multiple myeloma cells (NCT04126200). These clinical studies suggest that the elevated sBCMA levels seen in patients with advanced relapsed/refractory multiple myeloma interfere with BCMA-targeting antibodies binding to BCMA on tumor cell surface and thus impair therapeutic efficacy. In this study, we demonstrated that STI-8811 maintained cytotoxic activity in the presence of sBCMA at a level approximately 10-folds higher than levels sufficient to inhibit the belantamab mafodotin biosimilar, including at concentrations exceeding those measured in patients with relapsed/refractory multiple myeloma (>1,000 ng/mL). The resilience of STI-8811 against inhibition by sBCMA demonstrates enhanced clinical potential over the leading clinical candidate in patients with advanced relapsed/refractory multiple myeloma. Further investigation on this end would provide a better idea on the impact of sBCMA toward the therapeutic effect of ADCs.

Multispecies stability studies indicated that STI-8811 is stable in plasma for more than 21 days (>99% intact), which may contribute to the profound and long-lasting *in vivo* performance observed. Complete tumor regression and durable responses were widely observed after STI-8811 treatment in BCMA-high and BCMA-mid s.c. and disseminated models. In all cases, STI-8811 demonstrated greater efficacy and duration of tumor regression compared with the belantamab mafodotin biosimilar.

Although malignant PCs primarily reside in the BM, a subset of patients develops soft-tissue plasmacytomas arising from bone lesions or in extramedullary sites, including soft tissues and organs. Among patients with newly diagnosed and recurring multiple myeloma, 7% to 34.4% have paraskeletal lesions. Incidence of extramedullary lesions in patients with newly diagnosed multiple myeloma is 0.5% to 4.8% and is more common in relapsed/refractory disease (3.4%–14%; refs. 33–35). These soft-tissue plasmacytomas are characterized by increased proliferation, evasion of apoptosis, and resistance to therapies, including BCMA-targeted belantamab mafodotin, translating to poorer patient outcomes (33, 34, 39–41). In models of large solid tumors more closely resembling the size of plasmacytomas, STI-8811 demonstrated excellent tumor inhibition and complete tumor eradication without recurrence, outperforming the belantamab mafodotin biosimilar under the same conditions. The enhanced efficacy in large solid tumors may be attributed to a combination of features unique to our ADC: (i) the fast-on/fast-off binding kinetics reduce the impact of competitive binding to sBCMA in circulation, (ii) the more transient binding of our antibody to BCMA may allow STI-8811 to diffuse deeper into the tumor rather than irreversibly binding the first available BCMA receptor on the tumor periphery, and (iii) the bystander effect allows improved penetration of the payload into adjacent and inner tumor cells.

Clinical outcomes in patients with relapsed/refractory multiple myeloma after treatment with immunotherapies are characterized by heterogenous patient responses that can be attributed to interpatient and intratumor heterogeneity (42–44). Spatial and subclonal heterogeneous BCMA expressions allow for immunoediting of BCMA-high populations, leading to relapse characterized by BCMA-low or BCMA-null tumors (30, 45–48). The bystander cytotoxicity of STI-8811 against BCMA-negative tumor cells in the heterogeneous tumor context places STI-8811 in a unique position to address tumor heterogeneity and indicates that STI-8811 may be able to reduce the risk of disease recurrence.

In murine and cynomolgus models, we observed favorable toxicity over a relatively large therapeutic window. In our GLP toxicity studies, we observed toxicities consistent with those of auristatin payloads, including decreases in red blood cell, white blood cell, and platelet counts indicative of BM toxicities. All these changes recovered or trended toward recovery within the observation window following repeat dosing. Mild single-cell necrosis/mitosis of the corneal epithelium of the eyes was noted in female animals following treatment with STI-8811. The toxicity profile of STI-8811 is consistent with other BCMA-targeting ADCs and was well tolerated at and above therapeutic dose levels.

Overall, STI-8811 is a BCMA-targeting ADC with unique binding properties that demonstrates excellent tumor growth inhibition and regression under conditions that challenge current clinical BCMA-targeting ADCs while maintaining low toxicity and a favorable safety profile. We believe that STI-8811 warrants continued investigation in clinical studies given its ability to effectively repress tumors that are refractory to current therapies.

## Supplementary Material

Supplementary Table 1 Table S1. Surface BCMA expression level and corresponding STI-8811 cytotoxicity EC50 in 16 tumor cell lines

Supplementary Figure 1 Figure S1. Quantitative STI-8811 and J6M0-mc-MMAF internalization as measured by flow cytometry in RPMI8226 cell line expressing moderate BCMA levels (n = 2). Signal is measured following protease-K treatment to remove membrane bound antibody. Antibodies internalized per cell is calculated as mAb-Alexafluor 647 signal normalized to fluorophore:mAb ratio calibrated against quantitative fluorescent standard beads. Data represent mean ± SD

Supplementary Figure 2 Figure S2. Long-term tumor suppression study of repeat dosing of STI-8811 in NCI-H929 s.c. xenograft model. SCID-Beige (n = 7) mice were treated i.v. with 4 doses of indicated molecules at biweekly regimen. Data represent mean ± SD
