# Supplementary material for: Preclinical Evaluation of STI-8811, a Novel Antibody–Drug Conjugate Targeting BCMA for the Treatment of Multiple Myeloma
Source: Cancer Res Commun. 2024 Oct 11;4(10):2660–72. doi: 10.1158/2767-9764.CRC-24-0413 (PMC11467701; doi:10.1158/2767-9764.CRC-24-0413)
Supplement: Supplementary Table 1 — Table S1. Surface BCMA expression level and corresponding STI-8811 cytotoxicity EC50 in 16 tumor cell lines [file crc-24-0413_supplementary_table_1_suppst1.pdf]

Cell Line	Tumor Type	Avg BCMA receptors per cell	EC_{50} (nM)
			STI-8811
NCI-H929	Multiple Myeloma	24777	0.34
MM.1R	Multiple Myeloma	12077	2.07
OPM-2	Multiple Myeloma	9794	1.11
RPMI8226-GFP-Fluc	Multiple Myeloma	7972	39.97
MDA-MB-468	Adenocarcinoma	6793	484.00
U266	Multiple Myeloma	5837	0.53
RPMI8226	Multiple Myeloma	5786	116.90
MM.1S	Multiple Myeloma	5073	6.12
Daudi	Burkitt's Lymphoma	2456	258.55
K562	CML	1777	>1000
Ramos	Burkitt's Lymphoma	1591	87.94
ARH-77	Multiple Myeloma	1449	390.00
SkBr-3	Adenocarcinoma	1292	119.00
SU-DHL-1	Lymphoma	1226	268.80
HDLM-2	Hodgkin's Lymphoma	982	251.60
L-540	Hodgkin's Lymphoma	442	465.07

Table S1.

Surface BCMA expression level and corresponding STI-8811 cytotoxicity EC_{50} in 16 tumor cell lines.
