# Supplementary material for: Preclinical Evaluation of STI-8811, a Novel Antibody–Drug Conjugate Targeting BCMA for the Treatment of Multiple Myeloma
Source: Cancer Res Commun. 2024 Oct 11;4(10):2660–72. doi: 10.1158/2767-9764.CRC-24-0413 (PMC11467701; doi:10.1158/2767-9764.CRC-24-0413)
Supplement: Supplementary Figure 1 — Figure S1. Quantitative STI-8811 and J6M0-mc-MMAF internalization as measured by flow cytometry in RPMI8226 cell line expressing moderate BCMA levels (n = 2). Signal is measured following protease-K treatment to remove membrane bound antibody. Antibodies internalized per cell is calculated as mAb-Alexafluor 647 signal normalized to fluorophore:mAb ratio calibrated against quantitative fluorescent standard beads. Data represent mean ± SD [file crc-24-0413_supplementary_figure_1_suppsf1.pdf]

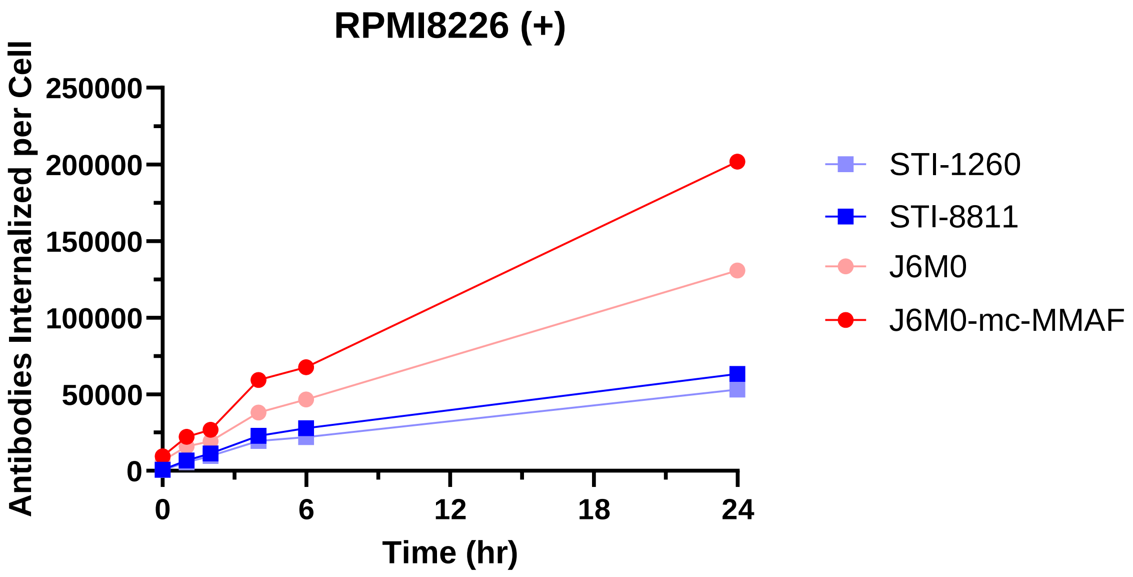

Figure S1.
Quantitative STI-8811 and J6M0-mc-MMAF internalization as measured by flow cytometry in RPMI8226 cell line expressing moderate BCMA levels (n=2). Signal is measured following protease-K treatment to remove membrane bound antibody. Antibodies internalized per cell is calculated as mAb-Alexafluor 647 signal normalized to fluorophore:mAb ratio calibrated against quantitative fluorescent standard beads. Data represent mean ± SD.
