# Supplementary material for: Preclinical Evaluation of STI-8811, a Novel Antibody–Drug Conjugate Targeting BCMA for the Treatment of Multiple Myeloma
Source: Cancer Res Commun. 2024 Oct 11;4(10):2660–72. doi: 10.1158/2767-9764.CRC-24-0413 (PMC11467701; doi:10.1158/2767-9764.CRC-24-0413)
Supplement: Supplementary Figure 2 — Figure S2. Long-term tumor suppression study of repeat dosing of STI-8811 in NCI-H929 s.c. xenograft model. SCID-Beige (n = 7) mice were treated i.v. with 4 doses of indicated molecules at biweekly regimen. Data represent mean ± SD [file crc-24-0413_supplementary_figure_2_suppsf2.pdf]

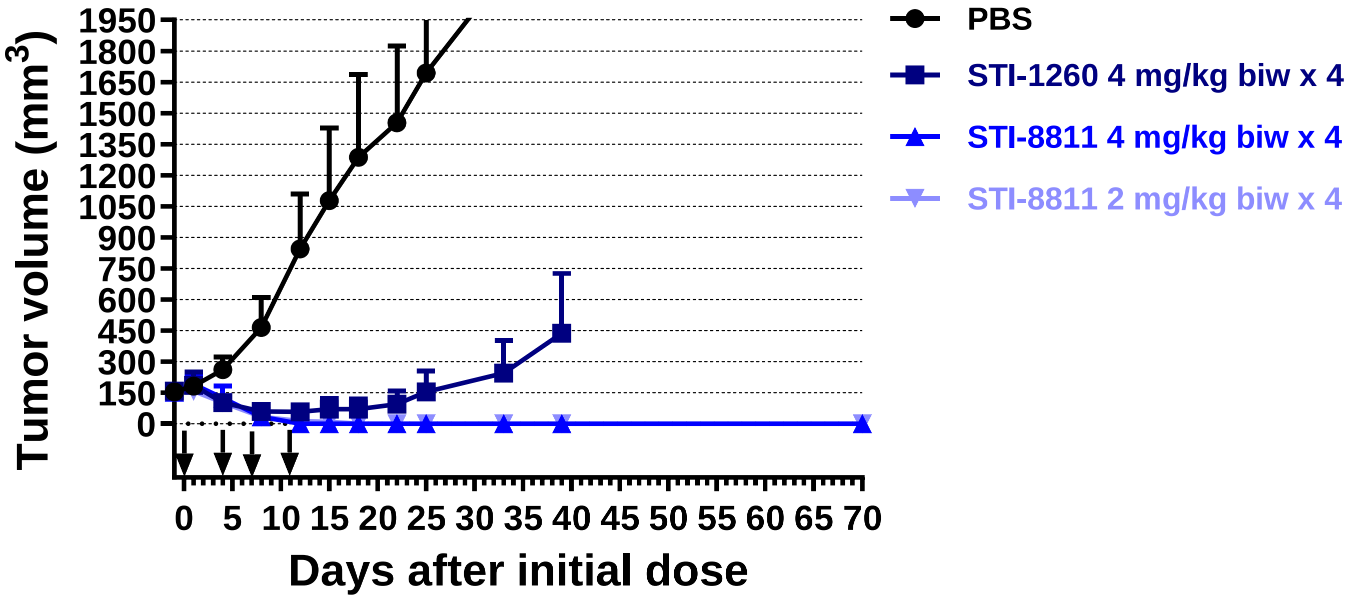

Figure S2.

Long-term tumor suppression study of repeat dosing of STI-8811 in NCI-H929 s.c. xenograft model. SCID-Beige (n=7) mice were treated i.v. with 4 doses of indicated molecules at biweekly regimen. Data represent mean ± SD.
